# From high school to hospital: how early exposure to healthcare affects adolescent career ideas

**DOI:** 10.5116/ijme.5801.f2cc

**Published:** 2016-11-06

**Authors:** Brandon Muncan, Nomrota Majumder, Nicolae Tudose

**Affiliations:** 1 Queens High School for the Sciences at York College, Jamaica, New York, USA; 2 University of Medicine and Pharmacy Timisoara, Romania

**Keywords:** High school to hospital, early exposure, healthcare, adolescent career

## Introduction

Exposure to various healthcare fields is crucial to the development of career interests for adolescents and young adults who plan to enter the world of medical practice. Globally, the number of opportunities for high school students to participate in research and clinical shadowing (direct observation of medical or paramedical practice) are limited.^1^ Recently however, many university programs and community hospitals in the United States and around the world have initiated interest programs for such students, allowing them to see, first-hand, the daily lives of health professionals. With the advent of specialized high school volunteering programs within hospitals and medical centers, teenage interest in pursuing a medical education has increased.^2^

### Volunteering activities and adolescent development

In general, volunteering at a young age has shown to improve educational pursuits, and to boost a student’s moral consideration.^3^ Involvement in organized activities, particularly in interpersonal settings correlates with “positive development” in the classroom and at home.^3^ Participation in any volunteering program helps an adolescent learn responsibility and empathy, alongside relationship-building and community connection.^3^ For the many students who consider entering the healthcare field, early exposure to careers and professionals that collaborate to treat patients is vital to a well-rounded education. As such, student involvement in hospital-organized programs is encouraged for anyone interested in medicine.

### Clinical and clerical experience

Volunteering programs oftentimes allow high school and undergraduate students to assist clerical and administrative staff with their work, thereby encouraging a greater appreciation and respect for all members involved in healthcare delivery. Responsibilities fall well within the skillset of a student, most often involving data input, charting, liaison, transportation, and informational duties.  Whereas many students begin clinical experience during graduate school, newer programs give younger students the option of clinical volunteering: assisting nurses, nurse practitioners, physician assistants and physicians in the tasks of caring for patients. As corroborated by past studies,^1^^,^^4^ early exposure to healthcare increases the ability of a student to “integrate practice and theory,” which evidently contributes to increased interest in the medical field.^5^ Students have the opportunity to witness, learn, and gain an understanding of multiple healthcare perspectives, which foster a holistic view of patient treatment. In addition to volunteering duties, participating students are encouraged to ask questions about topics and fields that pique their respective interests, contributing, thus to a growing interest in medicine. All-the-more, volunteers participate in seminars and lectures about bioethics, patient confidentiality, and safety protocol, which broaden exposure to the reality of medical care and brings students interested in health to a highly-informed platform. With the information and experience accumulated as a high school and undergraduate volunteer, a pupil becomes more confident about pursuing a healthcare track.

## Conclusions

As students, teachers, and mentors, we cannot underestimate the importance of early exposure for adolescents interested in medicine. High school and undergraduate students, through volunteering programs not only pursue their medical interest and learn about human biology at an early age, but also understand the importance of strong character. By shadowing medical professionals, volunteers have the opportunity to expand their horizons and explore the different components of an integrated health system. Overall, exposure to the everyday lives of medical professionals gives students insight into the responsibilities of healthcare professionals, and the enormous roles that such individuals play in the lives of patients.

By assisting medical staff with clerical tasks, students gain a sense of duty and realize the importance of giving back to community. By communicating and helping patients directly, volunteers not only create a cheerful atmosphere that improves patients' hospital stays, but also aid in the development of social skills necessary to build lasting connections with colleagues, mentors and the community.^3^ Evidently, increased high school and undergraduate student participation in hospital-based volunteer programs will produce a bright set of future physicians, physician's assistants, and nurses with promising intellectual and interpersonal qualities.

### Conflicts of Interest

The authors declare that they have no conflict of interest. 

## References

[1] Alqaryan SK (2016). A new approach to improving undergraduate research.. Int J Med Educ.

[2] Burgoyne LN, O'Flynn S, Boylan GB (2010). Undergraduate medical research: the student perspective.. Med Educ Online.

[3] Gardner M, Roth J, Brooks-Gunn J (2008). Adolescents' participation in organized activities and developmental success 2 and 8 years after high school: do sponsorship, duration, and intensity matter?. Dev Psychol.

[4] Khabaz Mafinejad M, Mirzazadeh A, Peiman S, Khajavirad N, Mirabdolhagh Hazaveh M, Edalatifard M, Allameh SF, Naderi N, Foroumandi M, Afshari A, Asghari F (2016). Medical students' attitudes towards early clinical exposure in Iran.. Int J Med Educ.

[5] Blanchard JA. Hospital volunteers: a qualitative study of motivation. The International Journal of Volunteer Administration. 2006;24(2):31-40.

